# Analysis of Phubbing Among University Students: A Study of Its Prevalence, Incidence Factors and Predictors

**DOI:** 10.3390/ejihpe15100201

**Published:** 2025-10-03

**Authors:** Pablo-César Muñoz-Carril, Inés M. Bargiela, Iris Estévez, Mónica Bonilla-del-Río

**Affiliations:** 1 Department of Pedagogy and Didactics, Faculty of Teacher Training, University of Santiago de Compostela, 27002 Lugo, Spain; 2 Department of Applied Didactics, Faculty of Teacher Training, University of Santiago de Compostela, 27002 Lugo, Spain; ines.mosquera.bargiela@usc.es; 3 Department of Pedagogy and Didactics, Faculty of Education Sciences, University of Santiago de Compostela, 15782 Santiago de Compostela, Spain; iris.estevez.blanco@usc.es; 4 Department of Education, Faculty of Education, Universidad de Cantabria, 39005 Santander, Spain; monica.bonilla@unican.es

**Keywords:** phubbing, smartphone, university, students, gender, age, academic performance, connection frequency

## Abstract

The ubiquitous presence of smartphones has led to new phenomenon such as “phubbing” (the act of ignoring one’s immediate surroundings in favor of using a mobile phone). This behavior has become increasingly common among university students, making it an important subject of study due to its potential negative impact on learning environments. The aim of the present study is to analyze the prevalence of phubbing among university students, the existence of significant differences as a function of specific sociodemographic variables (such as gender, age, academic performance, and connection frequency), and, lastly, the predictive capacity of these elements with the different levels of phubbing experienced. The sample was composed of 1121 Spanish university students, and the instrument selected for the collection of data was the Phubbing Scale, which was divided into three factors, “attachment to the mobile phone”, “communication disturbance”, and “smartphone obsession”, through different validity and reliability tests. The results indicated a moderately high prevalence of phubbing among the population studied. Likewise, statistically significant differences were identified at a multivariate level in the three dimensions. Lastly, it is notable that the frequency of smartphone usage significantly and positively predicted the three dimensions of phubbing.

## 1. Introduction

The incorporation of digital technologies in educational contexts has significantly transformed teaching and learning processes over the past two decades. Smartphones, tablets, and other mobile devices have enabled new forms of information access, communication, and content creation, while promoting flexible, student-centered learning (Zou et al., 2025). However, this technological integration has also introduced new challenges that affect the quality of educational interactions. Among them, phubbing has emerged as a behavior with potentially disruptive consequences in academic environments.

Phubbing can be defined as the act of ignoring other people in our immediate surroundings because we are focused and absorbed due to the use of a mobile connection (Blanca & Bendayan, 2018). Phubbing has become increasingly relevant in educational research, although initially it was conceptualized within social and relational frameworks. In classrooms emphasizing collaborative learning and active participation, phubbing can undermine students’ capacity for sustained attention and meaningful interaction. Recent studies have highlighted that such digital interruptions are associated with reduced academic engagement, emotional detachment, and even social withdrawal (Guerra Ayala et al., 2025). International policy frameworks have increasingly acknowledged these challenges. UNESCO’s (2021) Strategy on Technological Innovation in Education advocates for a humanistic approach to digital transformation, emphasizing that technology should enhance, not hinder, the learning experience. Similarly, the Digital Well-being Hub (OECD, 2021) highlights the importance of understanding how digital technologies affect well-being, gathering people’s experiences and statistical data to guide informed decisions and healthy tech use. These frameworks underscore the necessity of addressing behaviors like phubbing to promote holistic student development.

The study of phubbing in educational contexts aligns with broader concerns about digital dependency and its effects on cognitive, emotional, and social functioning. Theoretically, phubbing can be examined through Self-Determination Theory (Deci & Ryan, 2000), which posits that satisfying basic psychological needs (e.g., autonomy, competence, and relatedness) is fundamental for self-motivation and well-being. In educational settings, these needs are met through engagement, recognition, and positive social interaction, all of which may be compromised when students or instructors engage in phubbing behaviors. Extending this lens, Self-Determination Theory (SDT) further suggests that when these basic needs are thwarted in class (e.g., feelings of social disconnection or lack of competence), students may seek compensatory engagement via the smartphone as an immediate source of stimulation, control, or relatedness—thereby increasing the likelihood of phubbing in face-to-face contexts.

Furthermore, phubbing intersects with contemporary debates about the attention economy, digital well-being, and the pedagogical design of learning environments. Educational approaches relying on constructivist or socio-cultural learning models, which emphasize interaction, co-construction of knowledge, and dialogical engagement, are particularly vulnerable to the relational disruptions caused by phubbing. For instance, Koc and Caliskan (2022) demonstrated that instructor phubbing reduces student motivation and perceived engagement. From an attention economy perspective, smartphone ecosystems are deliberately engineered to capture scarce cognitive resources (e.g., via notifications, variable reinforcement, and seamless app-switching). Converging evidence shows that even the mere presence of one’s phone can reduce available cognitive capacity and bias attention away from the task at hand (Ward et al., 2017), which helps explain why phone-related cues so readily intrude on classroom interaction and learning.

Although conceptually related to adjacent constructs, phubbing maintains a distinct profile. Nomophobia, for example, refers to the anxiety or discomfort that arises when individuals are unable to access their mobile phone (Yildirim & Correia, 2015; León-Mejía et al., 2021; Muñoz-Carril & Dans Álvarez de Sotomayor, 2025). In a similar vein, Fear of Missing Out (FoMO) denotes the pervasive apprehension that others might be engaging in rewarding experiences in one’s absence, often leading to compulsive monitoring of online interactions (Przybylski et al., 2013; Wegmann et al., 2017). Although both nomophobia and FoMO are strongly associated with problematic smartphone use, they mainly capture underlying emotional and motivational vulnerabilities. Phubbing, by contrast, is specifically characterized by the behavioral displacement of face-to-face interaction through smartphone engagement. Clarifying this distinction underscores phubbing’s particular relevance for understanding how digital habits disrupt social presence and academic participation.

Given the pervasiveness of mobile phone use among university students, understanding phubbing also requires a multidimensional approach that considers individual, contextual, and cultural factors. This includes the influence of personality traits, academic stress, social norms, and digital habits. Sert et al. (2023) emphasize how chronotype and personality interact with gender to predict phubbing, while Guerra Ayala et al. (2025) link phubbing and nomophobia to sleep disturbances and mental health indicators such as stress and anxiety. In the Spanish context, recent evidence documents both high prevalence and distinctive profiles of phubbing among adolescents and university students and shows robust associations with problematic Internet/smartphone use and indicators of psychological adjustment (Barbed-Castrejón et al., 2024; Villalba Palacin et al., 2025).

Recent research on phubbing among university students reveals a complex interplay between digital behaviors, emotional well-being, and academic life. The ongoing debate around phubbing has shifted in recent years from viewing it as a mere social nuisance to understanding it as a multifaceted psychological behavior that both reflects and shapes students’ emotional and social experiences. Among university students, who face high digital connectivity demands and significant academic and social pressures, this behavior has become especially salient.

A growing body of studies illustrates how phubbing is closely intertwined with emotional vulnerabilities. For instance, Tufan et al. (2025) underscore that Fear of Missing Out (FOMO) and smartphone addiction are significant predictors of phubbing among students. These findings reinforce the cyclical dynamic wherein digital overuse, driven by social and academic anxieties, exacerbates emotional disconnection from immediate interactions.

This tendency appears particularly marked in students struggling with social anxiety. Oral and Karakurt (2025) demonstrated that socially anxious individuals often turn to smartphones as an escape mechanism during in-person encounters. Ironically, this strategy intensifies feelings of alienation and deepens dependence on mediated communication. This supports the view that phubbing is not merely a breach of etiquette but a coping behavior, albeit one with harmful consequences.

Psychological traits also influence students’ susceptibility to phubbing. According to Erzen et al. (2019), individuals high in neuroticism and low in conscientiousness are more likely to disengage from face-to-face interactions, suggesting that personality may predispose some students toward digital withdrawal. Complementing this idea, Bitar et al. (2023) found that emotional intelligence and self-esteem can buffer the negative emotional consequences associated with phubbing.

Adding to this body of work, Bitar et al. (2023) associated frequent phubbing with reduced psychological well-being and increased emotional detachment. Their research suggests that phubbing may serve as a form of avoidance, gradually weakening students’ capacity for meaningful connection and long-term relationship satisfaction. Together, these studies depict phubbing not as an isolated habit, but as a symptom of broader psychosocial dynamics in higher education. Consequently, effective interventions will need to address both emotional and behavioral dimensions, fostering resilience, digital literacy, and support structures for students navigating increasingly hybrid learning environments.

Beyond its emotional impact, phubbing also affects academic performance and the classroom dynamic. The disruption it causes extends beyond occasional distraction, interfering with core cognitive processes and eroding the social foundations of learning. Focusing specifically on intensive smartphone use in instructional settings, experimental and classroom studies show that phone-driven multitasking fragments attention, reduces note quality and retention, and lowers task performance (Junco, 2012; Kuznekoff et al., 2015). Complementary, attention economy evidence indicates that even the mere presence of a smartphone taxes working memory (Ward et al., 2017). Consistent with this, recent studies show that intensive smartphone use can undermine academic adjustment and performance; among first-year undergraduates, problematic mobile phone use mediates the adverse impact of cumulative ecological risk on academic adjustment, highlighting particular susceptibility during the transition to university (Yu et al., 2025). Meta-analytic and longitudinal evidence also links smartphone addiction/overuse to lower academic achievement, partly via academic anxiety and attentional costs (Zhang & Zeng, 2024; Paterna et al., 2024; Hsieh, 2025).

Importantly, phubbing does not only affect individual learning. It also shapes the social climate of the classroom. Pérez-Juárez et al. (2023) found that students perceive digital distractions as especially disruptive during collaborative activities like lab work. These distractions aren’t limited to peers. Research by Koc and Caliskan (2022) highlights the demotivating effect of instructor phubbing, in which educators themselves disengage during class, weakening instructional relationships.

These findings raise concerns for collaborative and constructivist learning models, where mutual presence and engagement are critical. When either students or instructors disengage through phubbing, opportunities for inclusive dialogue diminish and participation gaps may widen, especially among more vulnerable students. Addressing these challenges requires more than device bans; it calls for a cultural shift that prioritizes presence and models healthier digital behaviors.

In addition to academic settings, phubbing’s influence extends into daily routines, affecting sleep, mental health, and lifestyle patterns. Several studies show that the compulsive need to remain connected can interfere with students’ ability to disconnect and recover. For instance, a study by Guerra Ayala et al. (2025) linked phubbing with poor sleep quality and disrupted cycles, implicating it in diminished overall well-being.

At the psychological level, Bitar et al. (2023) reported that phubbing correlates with heightened symptoms of anxiety, stress, and depression. These conditions may not only be consequences of phubbing but also contributing factors, creating a feedback loop where students use digital distraction to cope with distress—only to find their symptoms worsened through emotional fragmentation and overstimulation.

Oral and Karakurt (2025) interpret this phenomenon as part of a broader pattern of emotional avoidance. Similarly, Aydin et al. (2024) introduce the concept of “digital obesity” to describe how overconsumption of digital content contributes to reduced physical activity, diminished social ties, and increased stress, all of which further degrade well-being. These findings underscore the importance of holistic interventions that not only target digital habits but also address the psychological stressors driving them.

Likewise, phubbing does not manifest uniformly across contexts. Its expressions and motivations are shaped by cultural norms, gender, and personal disposition. For instance, Barbed-Castrejón et al. (2024) show that phubbing behaviors differ across educational stages and cultural environments, with university students displaying distinct patterns shaped by autonomy, peer dynamics, and academic demands.

Gender differences also play a notable role. Escalera-Chávez et al. (2020) found that male university students exhibit slightly higher phubbing levels than their female counterparts. Sert et al. (2023) further explore how personality and chronotype influence phubbing tendencies, moderated by gender, suggesting that motivations and triggers differ between men and women.

Aside from gender, other sociodemographic variables have shown significant associations with phubbing among higher education students. In this sense, many studies have pointed out that age can play a relevant role, with younger students tending to manifest a greater use of the mobile phone in social situations, perhaps due to a higher digital exposure at early stages of life (Han et al., 2022). Likewise, the frequency of daily connections to the Internet has been identified as a key predictor of phubbing (Wolniewicz et al., 2021), as a greater digital exposure increases the probability of resorting to the use of the smartphone during moments of face-to-face interaction. In line with these patterns, studies in higher education consistently associate heavier smartphone/Internet use with more frequent phubbing, while reviews and field experiments converge on the view that attention captured by the device—sometimes by its mere presence—impairs both interaction quality and learning (Barbed-Castrejón et al., 2024; Matas-Terrón, 2025; Ward et al., 2017).

These variations in phubbing behaviors (shaped by educational stage, cultural environment, gender, age, frequency of digital use, etc.) point to the need for context-aware strategies. Universal awareness campaigns may fall short if they do not account for the cultural values, identity pressures, and social motivations that drive students’ digital behavior. A more nuanced, inclusive approach is essential for mitigating the social, emotional, and academic consequences of phubbing while supporting diverse student needs.

Considering these aspects, the aim of the present study is to deepen the understanding of the phubbing phenomenon in the university context. More specifically, the following research objectives were defined:

- To discover the prevalence of phubbing among university students.
- To analyze the differences in the dimensions of phubbing (“attachment to the mobile phone”, “communication disturbance”, and “smartphone obsession”), as a function of the sociodemographic variables such as gender, age, daily frequency of Internet connection, and academic performance.
- To examine the predictive ability of the sociodemographic variables gender, age, connection frequency, and academic performance, on each of the dimensions of phubbing.

Guided by SDT and attention economy accounts and aligned with prior trends, we advance the following directional hypotheses:

- **H1 (Usage).** Greater daily Internet connection time will be positively associated with higher overall levels of phubbing.
- **H2 (Academic performance).** Lower academic performance will be associated with higher overall levels of phubbing.
- **H3 (Gender).** Gender differences will emerge across the three phubbing dimensions (attachment to the mobile phone; communication disturbance; and smartphone obsession).
- **H4 (Age).** Younger students will exhibit higher overall levels of phubbing than older students.

The rationale for these hypotheses is as follows. For H1 (Usage), heavy daily connectivity increases opportunities for online activities to encroach on in-person interactions and fosters habitual checking; in Spanish samples, phubbing correlates positively with problematic Internet/smartphone use (Barbed-Castrejón et al., 2024), and a systematic meta-analysis identified problematic use patterns among the strongest phubbing predictors (Arenz & Schnauber-Stockmann, 2023). From an attentional standpoint, the mere presence of one’s smartphone can deplete cognitive resources (Ward et al., 2017), making device-directed shifts of attention more likely during face-to-face encounters. Regarding H2 (Academic performance), experimental and naturalistic classroom studies show that intensive smartphone use fragments attention and undermines learning processes such as note-taking and retention (Junco, 2012; Kuznekoff et al., 2015); together with the attention economy account (Ward et al., 2017), this provides a plausible mechanism linking higher phubbing with lower academic achievement (Chen et al., 2024). With respect to H3 (Gender), prior work suggests dimension-specific patterns: evidence points to relatively higher smartphone obsession among women (greater preoccupation/dependency) and equal or higher attachment to the phone/communication disturbance among men (more overt, interaction-disruptive use), a distinction consistent with gendered phone-use motives (Chotpitayasunondh & Douglas, 2016; Roberts & David, 2016). Finally, for H4 (Age), younger cohorts—acculturated to constant connectivity—typically report higher phubbing than older students; meta-analytic evidence indicates small age-related decreases in phubbing alongside robust links with intensive smartphone/Internet use (Arenz & Schnauber-Stockmann, 2023; Matas-Terrón, 2025; Muñoz Carril et al., 2025b).

These hypotheses are consistent with prior evidence reporting gender and age-related variability in phubbing among university students, robust associations between heavy daily connectivity and phubbing, and indications that intensive in-class smartphone use may undermine academic adjustment and performance (Escalera-Chávez et al., 2020; Han et al., 2022; Sert et al., 2023; Wolniewicz et al., 2021).

Despite the growing scientific literature on phubbing in recent years (particularly among university students) empirical research on this phenomenon remains relatively scarce, and important research gaps persist. In particular, few studies have simultaneously quantified phubbing prevalence and examined multiple sociodemographic and academic predictors within a single, multidimensional framework—especially in the Spanish higher education context. To address this gap, the present study offers an integrated analysis in a large sample of Spanish university students, combining percentile-based prevalence estimates with tests of group differences and multivariate predictors across theoretically distinct dimensions of phubbing. This approach allows us to evaluate the above hypotheses in a coherent, theory-driven manner.

## 2. Materials and Methods

The research was conducted following a quantitative methodology, with an ex post facto non-experimental design. The survey method was used as the main technique for data collection (Hernández-Sampieri & Mendoza, 2018; McMillan & Schumacher, 2005). Given the aim of the study, a descriptive and correlational approach was utilized.

### 2.1. Participants and Sociodemographic Characteristics

Convenience sampling was used to collect the data, and the study included the participation of 1121 university students enrolled in a Single Degree, Double Degree, or Master’s Degree in 30 different subjects taught at a public university located in Northeastern Spain.

The collection of information was performed in two phases. In the first, a pilot study was conducted with the participation of 250 subjects, which allowed us to validate the data collection instrument at the level of content and construct. Then, during the second phase, the survey was given to individuals who were part of the final sample, composed of 871 students.

With respect to gender, 78.8% (*n* = 686) identified themselves as women, 21.0% (*n* = 183) as men, while 0.2% (*n* = 2) declared themselves and non-binary. It must be pointed out that due to the small size of the latter category, it was excluded from the inferential analyses, only focusing on the major groups of women and men to guarantee statistical consistency.

With respect to age, the participants were distributed in the following ranges: 38.1% (*n* = 332) were younger than 20 years old, 46.4% (*n* = 404) were aged between 20 and 24 years old, 9.6% (*n* = 84) were between 25 and 29 years old, and 5% (*n* = 51) were older than 30 years old.

With respect to academic year, 28.0% (*n* = 244) were enrolled in their first year, 33.3% (*n* = 290) in their second, 18.7% (*n* = 163) in their third, 10.8% (*n* = 94) in their fourth, 8.3% (*n* = 72) in their fifth (in double-degrees), and 0.9% (*n* = 8) in an official Master’s degree.

With respect to academic performance, which was measured using the average grade in a given degree, a mean of 7.35 (SD = 1.15) was obtained, out of a maximum possible score of 10. When categorizing this variable, it was observed that 2.2% (*n* = 19) of the students had a low performance (less than 5 points), 38.1% (*n* = 332) a medium performance (between 5 and 6.99 points), 44.9% (*n* = 391) a high performance (between 7 and 8.99 points), and 14.8% (*n* = 129) a very high performance (9 points or more).

With respect to the daily frequency of Internet connection, 46.6% (*n* = 406) indicated being connected for four hours or longer, 23.1% (*n* = 201) between three and four hours, 17.2% (*n* = 150) between two and three hours, 10.2% (*n* = 89) between one and two hours, and only 2.9% (*n* = 25) for less than an hour daily.

### 2.2. Instrument

An ad hoc questionnaire was designed that included an initial section related to sociodemographic data with questions about gender, age, the type of degree, the degree studied, the academic year they were enrolled in, the university, the mean grade obtained, the type of devices used, and the frequency of Internet connection, among other aspects. Likewise, the survey included diverse scales that intended to evaluate the use patterns and possible abuses by the university students of Information and Communication Technologies (ICT). Among these scales, the Phubbing Scale (PS) was used, which was originally created by Karadağ et al. (2015) and posteriorly translated, adapted, and validated to the Spanish context by Blanca and Bendayan (2018). The PS was structured with a total of 10 items distributed into five response options, from 1 (never) to 5 (always), with a higher score obtained indicating higher levels of phubbing.

In both the original study and the Spanish one, the authors identified a structure with two correlated factors: “communication disturbance” (items 1 to 5 in the Spanish version and items 1, 2, 3, 4, and 10 in the original version), and “smartphone obsession” (items 6 to 10 in the Spanish revision, and 5 to 9 in the original), with both versions showing adequate levels of internal consistency.

In the first study, a confirmatory factor analysis (CFA) was first performed, replicating the original structure proposed in the studies by Karadağ et al. (2015) and Blanca and Bendayan (2018). However, the fit indices obtained were not adequate. For this reason, a preliminary exploratory factor analysis was performed with a random subsample composed of 250 university students. As a previous step before its application, the Kaiser–Meyer–Olkin test was performed (KMO = 0.866), which indicated a good sample adequacy, as well as a Bartlett sphericity test, which was significant (χ^2^ = 1114.99; gl = 45; *p* < 0.001).

The process of implementation of the exploratory factor analysis, performed through the maximum likelihood method and oblique rotation (Oblimin), revealed a structure of three correlated factors that explained 65.83% of the total variance: the first factor explained 40.29%, the second 13.74%, and the third 11.80%. During this analysis, item 6 (“My mobile phone is always within my reach”) was eliminated, given that its factorial load was less than 0.40 (Kline, 2011).

Based on the EFA findings and the conceptual analysis of the items, a new confirmatory factor analysis (CFA) was performed on the complete sample of 871 university students (without the 250 subjects that were part of the EFA included in this analysis), using the estimator Diagonally Weighted Least Squares (DWLS), which is especially recommended for ordinal data (Jöreskog et al., 2001; Li, 2016; Şimşek & Noyan, 2012; Xia & Yang, 2019). This analysis confirmed a structure of three correlated factors (see Table 1) that were named: “Attachment to the mobile phone” (items 3 and 5, which reflect negative reactions perceived by other people due to the use of the mobile phone), “communication disturbance” (items 1, 2, and 4, referring to the interfering use of the mobile phone in face-to-face social situations), and “smartphone obsession” (items 7, 8, 9, and 10, which allude to the compulsive use and the emotional dependence with the smartphone).

On the other hand, the fit indices obtained were found to be very adequate (CFI = 0.994; TLI = 0.991; IFI = 0.994; NFI = 0.991; RMSEA = 0.046 [90%CI: 0.033–0.059]; SRMR = 0.046; GFI = 0.996). Likewise, the factorial loads were high and statistically significant (*p* < 0.001), with values between 0.52 and 0.92, as shown in Table 1, with an adequate convergent validity of the items with their respective factors observed. Similarly, the internal consistency was also high for the total scale (α = 0.78), as well as adequate for each factor separately. More specifically, a Cronbach’s alpha of 0.69 was obtained for Factor 1 (“attachment to the mobile phone”), 0.76 for Factor 2 (“communication disturbance”), and 0.62 for Factor 3 (“smartphone obsession”).

### 2.3. Process of Data Collection

This subsection describes the sampling frame, recruitment procedures, instruments employed, and ethical safeguards applied in the study. The data collection was performed through the application of the questionnaires, in-person and online, encompassing a total of eight centers that were part of the public university system in Northeastern Spain. These were: Faculty of Business Administration and Management, University School of Labor Relations, Higher Polytechnic School of Engineering, Faculty of Veterinary Medicine, and Faculty of Sciences, Faculty of Humanities, Faculty of Teacher Training, and Faculty of Educational Sciences.

The questionnaire application process was performed in a staggered manner throughout the three academic years, including the most recent one, 2023–2024. To ease the distribution of the questionnaire among the students, and to obtain the pertinent authorizations, the center’s management teams, the coordinators of the different degrees, and the Vice-rectorate of the campus were asked to collaborate. All of them were provided with detailed information about the aims of the study, along with a formal request for authorization to administer the questionnaire in person in the classrooms. Likewise, they were provided with a digital version of the questionnaire, through the Microsoft Forms platforms, so that it could be disseminated through the institutional virtual classrooms of the virtual campus.

At all times, the confidentiality and anonymity of the answers were guaranteed, in line with the ethics principles of research and the current regulations related to the protection of personal data. For this, the questionnaire included an explicit declaration that informed the participants that their collaboration was voluntary, anonymous, and confidential. The participants were also informed that there were not correct or incorrect answers, encouraging the students to answer sincerely and reflectively, depending on their own experiences and perceptions.

It must be pointed out that during the second phase of the study, the application of the questionnaire—backed by the authorization from the Ethics Committee (code USC 28/2025)—continued to take place during academic year 2024–2025 in order to broaden the sample and to allow for future longitudinal analyses. Given that the collection and analysis of these new data are still underway, the corresponding data from this phase are not included in the present study.

### 2.4. Data Analysis

To provide an answer to the objectives of the study, diverse statistical tests using the programs IBM SPSS (version 27) and JASP (version 0.19.3.0) were performed. The analyses were structured around descriptive measurements, including frequencies and percentages, as well as measurements of central tendency (mean) and dispersion (standard deviation), in order to examine the distribution of the answers per item and to estimate the level of prevalence of phubbing, both globally and differentiated by dimensions. For this, cutoff points were established based on percentiles (P15, P80, and P95), according to widely used criteria used in studies on the problematic use of technologies (León-Mejía et al., 2021).

Posteriorly, multivariate analyses of the variance (MANOVA) were performed to explore the differences in the three dimensions of phubbing (“attachment to the mobile phone”, “communication disturbance”, and “smartphone obsession”) as a function of the sociodemographic variables such as gender, age, daily frequency of Internet connection, and academic performance. This technique was particularly appropriate because it allows the simultaneous testing of differences across multiple dependent variables, offering a more comprehensive assessment of group effects and reducing the risk of inflated Type I error. As significant multivariate effects were found, univariate analyses of the variance were performed (ANOVA), as well as post hoc tests, to identify in what specific dimensions these differences were produced.

Multiple linear regressions analyses were performed to estimate the predictive power of the sociodemographic variables on each of the phubbing dimensions. This approach was selected as it makes it possible to assess the unique predictive contribution of each variable while controlling for the others, thereby clarifying their relative influence. In addition, regression models provide information about the proportion of explained variance, which offers a more precise understanding of the weight and explanatory capacity of each factor associated with phubbing in the university context.

Lastly, for the multivariate tests, we report Pillai’s Trace given its robustness to mild violations of multivariate assumptions. In univariate follow-ups, Levene’s tests were used to assess homogeneity of variances; where heteroscedasticity was indicated, results were verified with Welch-corrected ANOVA, yielding consistent conclusions. For multiple regression, we examined linearity and residual patterns; because deviations from normality and homoscedasticity are common with Likert-type outcomes in large samples, we confirmed in-text results using heteroscedasticity-robust (HC3) standard errors, which did not change inferential conclusions. Multicollinearity was assessed via variance inflation factors (VIF) and tolerance, with all VIF values < 3. We report effect sizes (partial η^2^ for ANOVA) and include 95% confidence intervals for descriptive means (Table 2) and for regression coefficients in the corresponding tables. Moreover, given the cross-sectional, observational design, all coefficients are interpreted as conditional associations rather than causal effects. The term “predictor” in regression is used in a statistical sense (i.e., contribution to explained variance), not to imply causation.

It should be noted that, to mitigate potential common method bias (CMB), the study design ensured anonymity and confidentiality, voluntary participation, varied item order, and administration in the absence of instructors (research staff only). Ex post, Harman’s single-factor test indicated no dominant method factor. Consistent with the theoretical specification, a three-factor solution was retained and corroborated by CFA for ordinal data (see CFA results reported elsewhere in the manuscript). As an additional post hoc check, none of the interfactor correlations approached the redundancy threshold of 0.90 (Attachment–Communication r = 0.45; Attachment–Obsession r = 0.37; Communication–Obsession r = 0.51), a pattern consistent with discriminant validity and not indicative of a dominant common method factor (Kline, 2023).

## 3. Results

### 3.1. Descriptive Analysis and Levels of Prevalence of Phubbing

Table 2 presents item-level descriptive statistics for the phubbing scale (frequencies, percentages, means, and standard deviations) and 95% confidence intervals for the means. For clarity, response categories were grouped as Never/Almost never, Sometimes, and Almost always/Always.

Overall, the response patterns suggest that university students generally downplay the interpersonal conflicts typically attributed to phubbing. The very high concentration of answers in the lower categories for items such as *“People complain about me dealing with my mobile phone”* or *“I think that I annoy my partner when I am busy with my mobile phone”* indicates that most participants do not perceive explicit signals of external disapproval regarding their mobile use. In contrast, behaviors reflecting routine availability and automatic checking (such as keeping the phone constantly within reach or checking it immediately upon waking up) emerged as the most normalized and frequent practices. These findings highlight a distinction between *externally disruptive* aspects of phubbing, which are perceived as rare, and *habitual/self-regulatory* dimensions, which appear widely integrated into students’ daily routines.

At the same time, the low endorsement of items associated with “smartphone obsession” (e.g., feeling incomplete without the phone or progressively increasing its use) suggests that participants do not generally identify with more problematic or addictive patterns. This aligns with the relatively low mean scores for these items, pointing to a self-perception of controlled smartphone use. However, the wide variability observed in the item concerning the reduction in time devoted to social, personal, or academic activities reflects heterogeneous profiles; while most students perceive minimal interference, a smaller group acknowledges a tangible impact of their phone use on daily functioning.

Regarding other aspects, and with the aim of estimating the prevalence of phubbing in the sample analyzed, the total score was calculated for each participant from the scale items. Posteriorly, and following a classification strategy widely used in research on behavioral addictive behaviors and the problematic use of technologies (León-Mejía et al., 2021; González-Cabrera et al., 2017; López-Fernández et al., 2013), cutoff values were established based on the 15, 80, and 95 percentiles of the total scores obtained in the sample.

The corresponding values for these percentiles were: P15 = 14, P80 = 25 and P95 = 31. Based on these thresholds, and according to the proposal by Moure-Rodríguez et al. (2019), four levels of the presence of phubbing were established (see Table 3).

As the data in Table 3 show, the results obtained indicate that phubbing is moderately widespread among university students, with 67.3% of the participants who could be considered frequent phubbers. On the other hand, a not inconsiderable 21.1% of the sample showed levels that could be considered worrying, as 15.5% were found to be in a situation of risk, while 5.6% obtained scores indicating a problematic use of the smartphone in interpersonal contexts. The category of occasional phubbing was the least frequent (11.6%), which suggests that the behavior of using the mobile phone in the presence of others is normalized, becoming relatively habitual, although only a minority experiences it with a high intensity.

A more detailed analysis, based on the three dimensions of phubbing identified in the confirmatory factor analysis (“attachment to the mobile phone”, “communication disturbance”, and “smartphone obsession”), as well as the cutoff points established using the 15, 80, and 95 percentiles, revealed, as shown in Table 4, that, in general, most of the students could be found within the frequent levels of use of mobile phones in interpersonal contexts. Nevertheless, it was also observed that a significant portion of the students have high levels of use that are risky or problematic, especially in the dimensions “communication disturbance” and “smartphone obsession”.

### 3.2. Multivariate Analysis of the Phubbing Dimensions as a Function of the Sociodemographic Variables

A multivariate analysis of the variance (MANOVA) was performed to identify the existence of statistically significant differences among the three dimensions of phubbing identified in the CFA (“attachment to the mobile phone”, “communication disturbance”, and “smartphone obsession”)—which act as dependent variables—with respect to other independent sociodemographic variables such as gender, age, Internet connection frequency (without taking into account academic or study tasks), and academic performance. All inferential results below are reported as associations between variables; no causal interpretation is intended.

#### 3.2.1. Gender Differences in Phubbing Dimensions

With respect to gender, Box’s M test provided a result of 15.20, gl = 6, *p* = 0.0188, which indicates a violation of the assumption of equality of the covariance matrices. For this reason, the test statistic Pillai’s Trace was used, considered to be more robust in light of a lack of homogeneity. The MANOVA performed revealed significant differences as a function of gender (Pillai’s Trace = 0.0252; F(3, 870) = 7.455, *p* < 0.001, η^2^ = 0.025), with the effect size being low. As shown in Table 5, men obtain higher means than women in the dimensions “attachment to the mobile phone” and “communication disturbance”, while women scored slightly higher in “smartphone obsession”.

#### 3.2.2. Age Group Differences in Phubbing Dimensions

As for age, Box’s M test showed the non-existence of the homogeneity of the covariances (Box’s M = 61.57, gl = 18, *p* < 0.001), so Pillai’s Trace was used for the analysis of multivariate significance of the main effects. The results obtained were statistically significant between the age groups (Pillai’s Trace = 0.0456; F(9, 868) = 4.459, *p* < 0.001, η^2^ = 0.046). Although the effect size was low, differences were observed in the three factors (Table 6), with the youngest students (younger than 20 years old) showing higher scores in “communication disturbance” and “smartphone obsession”.

#### 3.2.3. Differences by Daily Internet Connection Time

As for the daily Internet connection frequency, as no evidence was found of the lack of compliance with the homogeneity of variances assumption, the Wilks’ Lambda statistic was used. The MANOVA revealed significant differences between the groups (Wilks’ Lambda = 0.906; F(12, 2286) = 7.21; *p* < 0.001), with an effect size of η^2^ = 0.094, which indicates an important variation in the levels of phubbing (in the three dimensions), as a function of the frequency of Internet use.

As shown in Table 7, the phubbing levels of the university students increased as the daily Internet connection time increased, especially underlining the dimension “smartphone obsession”.

#### 3.2.4. Differences by Academic Performance

With respect to academic performance, Wilks’ Lambda was also used as the comparison statistic, with significant differences found between the levels of performance (Wilks’ Lambda = 0.960; F(9, 2105) = 3.98; *p* < 0.001), with a low effect size (η^2^ = 0.040).

As Table 8 shows, the means obtained according to the level of academic performance showed that students with a low performance tended to have higher scores in the phubbing dimensions. This trend was especially evident in “attachment to the mobile phone”, where an inverse relationship was observed between the use of the device and academic performance. Although the differences in “communication disturbance” and “smartphone obsession” were more discrete, the data suggests that a greater use of the mobile phone could be associated with a lower academic performance.

#### 3.2.5. Specific Group Differences and Effect Sizes

After identifying significant differences in the multivariate analysis, univariate analyses (ANOVA) were performed to examine the specific dimensions of phubbing in which these differences were found as a function of the sociodemographic variables considered.

With respect to gender, although the MANOVA analysis indicated statistically significant differences in this variable, the univariate analyses (ANOVA) did not reveal the existence of significant differences in any of the phubbing dimensions that were evaluated separately. More specifically, the results obtained were the following: “attachment to the mobile phone” (F(3, 867) = 2.430, *p* = 0.064, η^2^ = 0.008), “communication disturbance” (F(3, 867) = 0.594, *p* = 0.619, η^2^ = 0.002), and “smartphone obsession” (F(3, 867) = 0.798, *p* = 0.496, η^2^ = 0.003).

With respect to age, significant differences were observed in the three dimensions, “attachment to the mobile phone” (F(3, 867) = 5.044, *p* = 0.002, η^2^ = 0.017), “communication disturbance” (F(3, 867) = 4.271, *p* = 0.005, η^2^ = 0.015), and “smartphone obsession” (F(3, 867) = 8.112, *p* < 0.001, η^2^ = 0.027), although the effect sizes were small. It must be pointed out that in every case, the younger participants (<20 years old) showed higher mean values.

As for the frequency of connecting to the Internet, significant differences were also observed in the three dimensions: “attachment to the mobile phone” (F(4, 866) = 5.281, *p* < 0.001, η^2^ = 0.024), “communication disturbance” (F(4, 866) = 3.628, *p* = 0.006, η^2^ = 0.016) and “smartphone obsession” (F(4, 866) = 5.276, *p* < 0.001, η^2^ = 0.024). The students who manifested being connected for more than four hours per day (without counting the time spent studying) obtained higher scores.

As for the ANOVA results obtained for academic performance, they indicated statistically significant differences in the three dimensions: “attachment to the mobile phone” (F(3, 867) = 7.477, *p* < 0.001, η^2^ = 0.025), “communication disturbance” (F(3, 867) = 3.872, *p* = 0.009, η^2^ = 0.013), and “smartphone obsession” (F(3, 867) = 4.503, *p* = 0.004, η^2^ = 0.015). The students with lower scores showed higher levels of phubbing, especially in the dimension “attachment to the mobile phone”.

### 3.3. Prediction of the Phubbing Dimensions Through a Multiple Linear Regression

To examine the predictive ability of the different sociodemographic variables on the three dimensions of phubbing, different multiple regression analyses were performed through the use of the block input method. In all the models, gender, age, daily frequency of Internet connection (excluding academic tasks) and academic performance were introduced as the predictors. The assumptions of normality of residues, lack of multicollinearity, and homoscedasticity, were verified, without substantial violations found.

In the dimension “attachment to the mobile phone”, the model was statistically significant (F(13, 857) = 4.467, *p* < 0.001), explaining 6.3% of the variance (R^2^ = 0.066). Within the predictors, gender had a significant effect, so that being a woman was associated with lower levels of attachment (β = −0.139, *p* = 0.018), indicating a higher emotional “attachment to the mobile phone” among men. Differences were also observed as a function of age, as the students younger than 20 years old showed a significantly higher score in this dimension (β = 0.117, *p* = 0.025). Likewise, the frequency of connection to the Internet showed a relevant weight. More specifically, connecting between 1 and less than 3 h daily was associated with lower levels of attachment than being connected for more than 4 h (β = −0.271, *p* < 0.001 and β = −0.174, *p* = 0.005, respectively). Lastly, academic performance also acted as a predictor. Thus, the students with a lower performance (mean grade < 5) obtained higher attachment scores (β = 0.113, *p* = 0.007), as did those who had a medium performance (β = 0.089, *p* = 0.030), as compared to those who had a very high performance.

In the dimension “communication disturbance”, the model also reached statistical significance (F(13, 857) = 4.697, *p* < 0.001), explaining 6.7% of the variance (R^2^ = 0.070). However, in this case, the only variables that showed a significant influence were related to the frequency of Internet connection. More specifically, the students who indicated being connected for less than 4 h per day (either between 1 and less than 2 h, between 2 and less than 3 h, or between 3 and less than 4) scored significantly lower in this dimension as compared to those who were connected for more than 4 h (*p* < 0.01 in every case). On the contrary, no significant effects were found according to gender, age, nor academic performance. This implies that the analysis of linear regression showed that only the frequency of mobile phone use was a statistically significant predictor of this factor. This suggests that even though other variables such as age or academic performance could be associated with differences in groups, their ability to directly predict the level of “communication disturbance” was limited when the effects of the other variables of the model were controlled for.

Lastly, in the dimension “smartphone obsession”, the model showed a higher global fit out of all three (F(13, 857) = 7.539, *p* < 0.001), explaining 10.3% of the variance (R^2^ = 0.106). In this case, the women obtained higher scores than the men (β = 0.135, *p* = 0.034). A significant influence was also found for the variable age, with those older than 30 obtaining the lowest scores (β = −0.267, *p* = 0.019), as compared with the groups of younger individuals. Once again, the connection frequency was a solid predictor, as all the categories of a connection of less than 4 h daily showed obsession scores that were significantly lower as compared with those who were connected for more than 4 h (*p* < 0.01). In addition, academic performance once again showed a significant influence, with the students who had a high academic performance (grade ≥ 9) showing significantly lower scores in this dimension (β = −0.230, *p* = 0.017).

## 4. Discussion

Considering the differential diversity of the prevalence of phubbing, the divergences in the manifestation of this phenomenon according to sociodemographic factors, and the diverse harmful consequences associated with it, the study tries to shed light on some elements considered to be key in the comprehensive analysis of its development and its potential prevention (Arenz & Schnauber-Stockmann, 2023; Blanca & Bendayan, 2018).

Firstly (and providing an answer to the objective posed), the results obtained allowed us to discover the levels of prevalence of phubbing in the population of university students. In this way, it is underlined that this behavior is moderately widespread among higher education students. Close to 2 out of 3 students (67.3% of the sample) can be considered frequent phubbers; that is, they manifest these types of maladaptive behaviors habitually, without clear signs of risk. These numbers are aligned with (and even exceed) the indices of prevalence of phubbing shown in other studies in Spain (Barbed-Castrejón et al., 2024), but also in international contexts such as the USA (prevalence of 41.3%; Lo et al., 2022) or India (prevalence of 42.7%; Davey et al., 2018).

On the other hand, approximately 1 out of 5 students show levels that could be considered worrying. In more detail, 1 out of 6 students (15.5%) was found in a situation of risk, and almost 1 out of 18 (5.6%) showed a problematic use (severe) of the smartphone in interpersonal contexts. Although the levels reported in Turkish students was not reached, which referred to 12.7% of clinical or problematic phubbing (Ahmed et al., 2023), the values found are still worrying, and even more so considering the exponential growth and the negative repercussions associated with the phenomenon (Bitar et al., 2023; Oral & Karakurt, 2025; Tufan et al., 2025).

In fact, the variables observed that provided higher mean indices were related to the reach at which the students have the phone, and that checking it is the first thing (or one of the first things) they do as soon as they wake up, suggesting the existence of an almost constant availability of the mobile phone, an aspect that could act as a facilitator of phubbing behaviors when increasing the opportunities of use during social interactions, which could also be associated with other phenomena such as nomophobia (Guerra Ayala et al., 2025; Muñoz-Carril & Dans Álvarez de Sotomayor, 2025) or FOMO (Tufan et al., 2025).

It is also important to point out that this phenomenon has been analyzed starting with the three factors that shape and characterize it: “attachment to the mobile phone”, “communication disturbance”, and “smartphone obsession” (see: Blanca & Bendayan, 2018; Karadağ et al., 2015). This allows us to have certainly specific and refined view of their degree of presence and the different ways that phubbing is manifested in the students. Thus, it is revealed that the dysfunctional use of the phone in social situations is certainly marked around “communication disturbance”. In this case, seven out of ten students, in a frequent manner, negatively alter their personal interactions with others due to being busy with their phones (and three out of ten identify it with a situation of risk or a problematic situation). In addition, with respect to the dimension “attachment to the mobile phone”, it was also observed that a significant portion—almost eight out of ten students (78.3%)—indicated thattheir close environment frequently complained and was annoyed by the lack of attention experienced due to checking their phone. This trend is maintained, although to a lesser degree, in the dimension “smartphone obsession”. In this case, six out of ten students provided information on frequent behaviors associated with prioritizing phone use over other activities, or the excessive concern for the smartphone, and two out of ten enter a risky or problematic level in this area.

Next, to provide an answer to the second objective of the study, some elements whose variability may create differences in the prevalence of phubbing and its dimensions were identified (i.e., Blanca & Bendayan, 2018; Karadağ et al., 2015). In this way, significant differences in the degree of manifestation of phubbing were observed, taking into account this multivariate space (that is, considering the linear combination of all the sociodemographic variables analyzed: age, gender, academic performance, and connection frequency). After the analysis of the differences for each of the independent variables specifically, it was concluded that: the students younger than 20 (the youngest ones) who connected with a frequency of more than four hours per day (without taking into account the time spent studying), with the lowest grades (the lowest academic performance), obtained the highest mean scores in all the phubbing dimensions analyzed.

The case of gender implies a complementary reflection, as the multivariate level shows differences indicating that men have a higher “attachment to the mobile phone” and a more substantial “communication disturbance”, and on their part, women were shown to be more “obsessed with the smartphone”. The fact is that this does not occur when using the variable gender alone. These findings suggest that the levels of phubbing in the sample studied do not significantly vary according to gender separately, which leads us to reject—at the univariate level—the third hypothesis formulated. This result challenges the conclusions from other recent studies, with university populations, which showed significant differences. For example, Escalera-Chávez et al. (2020) and Barbed-Castrejón et al. (2024) stated that men show higher values than women; the study by Anshari et al. (2016) and the one by Karadağ et al. (2015) revealed that female students showed higher rates of prevalence than their male peers.

Considering the divergence of the gender-related results, it is deduced that the focus of analysis can create this discrepancy. After the analysis of the variance and the multivariate analysis, it was concluded that the combination between the variables (gender, age, frequency of connection, academic performance, and perhaps others that were not addressed in this study) shows a significant pattern that allows us to differentiate the levels of phubbing between the groups. Therefore, the preventive or educational interventions directed towards reducing the presence of this phenomenon must be mainly centered on the profile of connectivity, age group, and academic performance of the students, with gender considered as contextual variable (that is, in interaction with others) being useful.

Going a step further and addressing the results emanating from the predictive ability of the sociodemographic variables (gender, age, connection frequency, and academic performance) in each of the phubbing dimensions, it is concluded that the percentage of variance explained by the models indicates a statistically significant yet moderate relationship. In this way, relevant information is offered that helps in the continuous construction of a theoretical framework about such a complex and multi-faceted phenomenon as phubbing. This, at the same time, helps to gauge the causality of its generation and evolution, considering the multidimensionality of factors that shape phubbing. In line with the recent meta-analysis work conducted by Arenz and Schnauber-Stockmann (2023), the results obtained indicate that in a global manner, all the variables studied predict the phubbing phenomenon.

Thus, it is observed that the profile of being male, younger than 20 years old, being connected for more than 4 h per day (without taking into account the time spent studying), and having a low to medium performance shows a higher propensity of “attachment to the mobile phone”. On its part, “smartphone obsession” is a variable predicted for the female group, with an age younger than 30 years old, with an Internet connection of more than 4 h, and an academic performance between low and medium. This indicates a constant worry or compulsive use of the mobile phone in this group.

Lastly, it must be underlined that in the third model, the connection frequency (more than four hours) was identified with the manifestation of the “communication disturbance”, discarding the predictive ability of the variables gender, age, or academic performance for this factor. This suggests that the impact of mobile phone use on communication interactions is a cross-cutting phenomenon among different student profiles and is more associated with the usage time (confirming the first hypothesis) thanwith personal or academic characteristics.

Thus, gender once again provides a differential result as compared to the literature reviewed (i.e., Escalera-Chávez et al., 2020, in which the male students exhibit slightly higher phubbing levels than their female counterparts, or Karadağ et al., 2015, who offer opposing results, indicating that women are more inclined to engage in phubbing.). In the findings obtained in the present study, gender only had an important role in combination with other variables. It is therefore insisted that future analysis of this variable in the phubbing phenomenon should not be performed in an isolated manner, but it would be important to consider it a contextual, mediating or moderating variable from a person-centered approach (Aydin et al., 2024).

With respect to the age of the participants, the findings are in line with the contributions by (Han et al., 2022), given that in most cases, significant differences exist and even a statistical prediction based on this variable. The younger groups showed a greater tendency of manifesting phubbing behaviors, which confirms the fourth hypothesis proposed.

On the other hand, academic performance also seemed to be a predictor of phubbing. The study by Baranova et al. (2023) provided information on a significant and negative correlation between both variables, which could point to academic performance as a causal or consequent element of phubbing. In our case, the predictive ability was observed, pointing to a low academic performance promoting the development of higher levels of “attachment” and “smartphone obsession”. It is therefore deduced that the students with a lower performance are more demotivated, not satisfied, or bored, thereby perceiving the use of the phone as entertainment, a source of constant and instant gratification, and/or an escape route. This result allows us to confirm the second hypothesis proposed. Nevertheless, this finding also leads us to consider and propose the (future) hypothesis that phubbing could also have negative effects on students’ academic performance (i.e., influence it in the opposite direction). Following the arguments by Lukose and Agbeyangi (2025), various elements of the education process could be compromised due to the compulsion with the smartphone and the disconnection from face-to-face situations. For this reason, further studies are encouraged that address the analysis of the bidirectional causality—in a feedback loop—of the relationship between academic performance and phubbing.

As the data are cross-sectional and self-reported, causal inferences are unwarranted. Bidirectional processes are plausible—for example, higher phubbing may co-occur with poorer academic adjustment, while academic difficulties may also be associated with increased phone-directed attention. In addition, residual confounding (e.g., personality traits, social connectedness, and academic stress) may partly account for the observed associations. Longitudinal and experimental designs are needed to clarify directionality and rule out alternative explanations.

The only variable studied that showed to have a predictive ability in all of the dimensions evaluated (in the three models) was the frequency of smartphone use (without taking into account the study or academic time). This result is directly aligned with that provided by Ergün et al. (2020). It can be inferred, therefore, that the longer individuals use their phone, the more they need to use it. Entering into an incremental loop can lead to digital addiction or digital obesity (Aydin et al., 2024). In fact, the latter term—understood as an excessive and unhealthy use of technologies—is associated, from a person-based approach, with phubbing, to conclude that individuals who combine both variables have a level of life satisfaction that is significantly lower than their peers with a low addiction (Aydin et al., 2024). This could indicate that some competencies, such as time management, emotional and behavioral self-regulation, as well as a high academic self-efficacy or adaptive and adjusted motivational orientation (Deci & Ryan, 2000), could be protective factors against more dysfunctional uses of mobile devices.

It is deemed necessary to allude to the limitations of the present study, mainly due to its cross-sectional nature, the type of sampling used, and the fact that all measures rely on self-reported questionnaires (which may be subject to social desirability bias). These conditions demand the prudent interpretation of the results obtained.

On the other hand, the factorial structure that emerged does not correspond to the original (Blanca & Bendayan, 2018; Karadağ et al., 2015), which implies an effort when comparing and contrasting the findings around three dimensions. This also directs us towards conducting future studies in order to create a more solid and comprehensive framework for the constitutive dimensions of phubbing. In addition, it is deemed important to perform multivariate regression analyses to construct structural models, or even to analyze latent classes in the prospective lines of work around phubbing in educational environments.

Findings could inform policy guidelines on digital literacy (macrofocus) and/or some guidelines to implement in the daily life of university students (microfocus). First and most significantly, considering that connection hours are—among the variables studied—the greatest predictor of phubbing behaviour, it is necessary to promote the integration of ‘screen-free moments,’ seeking leisure alternatives that do not involve the use of smartphones. Therefore, as a preliminary matter, the objective should be to develop skills to balance the use of technology autonomously and deliberately, as part of a structured, compulsory training programme integrated into the curriculum. It is also proposed to begin the training programme from early stages and to continue it during the university years. In this educational process, it would be important to inform people about the potential harms of phubbing, encouraging reflection, self-criticism and awareness. It would also be important to offer tools for managing screen time and resources for emotional and behavioural self-regulation when faced with the impulse or need to stay connected via smartphone.

In this regard, evidence demonstrates that psychoeducational interventions on digital hygiene significantly reduce time spent on social networks and improve wellbeing indicators such as sleep (Olivo-Martins-De-Passos et al., 2024). Furthermore, from an institutional and community perspective, it is essential to provide healthy leisure alternatives and foster spaces for active disconnection. Initiatives such as “screen-free days”, as previously mentioned, in university settings, which have been associated with improvements in concentration and reductions in stress (Kolhe & Naik, 2025), alongside programmes promoting physical activity (Brown et al., 2024), community urban gardens that enhance psychosocial wellbeing and healthy eating habits (Hume et al., 2022), or healthy cooking workshops that reduce the consumption of ultra-processed foods (Watanabe et al., 2024), represent effective and transferable strategies. Complementarily, interventions based on mindfulness and cognitive behavioural therapy have proven effective in reducing compulsive digital behaviours and improving emotional regulation (Sancho et al., 2018; Sudhir, 2018), reinforcing the possibility of their incorporation into university prevention programmes. To ensure sustainability and effectiveness, these initiatives require institutional support and the active engagement of the entire university community, so that they may be consolidated into educational and public health policies at national and international levels, thereby promoting healthier uses of technology among university populations (Muñoz Carril et al., 2025a).

## Figures and Tables

**Table 1:** Factor loads obtained in the confirmatory factor analysis (CFA) of the phubbing scale.

Items	Factorial Loads	Factors
Phu-3. People complain about me dealing with my mobile phone	0.75	Factor 1—Attachment to the mobile phone
Phu-5. I think that I annoy my partner when I’m busy with my mobile phone (or family, if you do not have a partner)	0.92	
Phu-1. My eyes start wandering on my phone when I’m together with others	0.83	Factor 2—Communication disturbance
Phu-2. I am always busy with my mobile phone when I’m with my friends	0.89	
Phu-4. I’m busy with my mobile phone when I’m with family	0.78	
Phu-7. When I wake up in the morning, I first check the messages on my mobile phone	0.52	Factor 3—Smartphone obsession
Phu-8. I feel incomplete without my mobile phone	0.77	
Phu-9. My mobile phone use increases day by day	0.79	
Phu-10. The time allocated to social, personal or professional activities decreases because of my mobile phone	0.68	

**Table 2:** Phubbing scale items and descriptive statistics (n = 871).

Item	Never & Almost Never		Sometimes		Almost Always & Always		Mean	95% CI (Lower)	95% CI (Upper)	SD
	*n*	%	*n*	%	*n*	%				
Phu-1. My eyes start wandering on my phone when I’m together with others	609	69.92	170	19.52	92	10.57	2.05	1.98	2.12	1.04
Phu-2. I am always busy with my mobile phone when I’m with my friends	788	90.47	68	7.81	15	1.72	1.45	1.4	1.5	0.74
Phu-3. People complain about me dealing with my mobile phone	795	91.27	52	5.97	24	2.75	1.30	1.25	1.35	0.73
Phu-4. I’m busy with my mobile phone when I’m with family	718	82.43	119	13.66	34	3.9	1.60	1.54	1.66	0.89
Phu-5. I think that I annoy my partner when I’m busy with my mobile phone (or family, if you do not have a partner)	763	87.6	63	7.23	45	5.17	1.43	1.37	1.49	0.88
Phu-6. My mobile phone is within my reach	154	17.68	131	15.04	586	67.28	3.84	3.75	3.93	1.29
Phu-7. When I wake up in the morning, I first check the messages on my mobile phone	268	30.77	127	14.58	476	54.65	3.41	3.31	3.51	1.49
Phu-8. I feel incomplete without my mobile phone	669	76.8	138	15.84	64	7.35	1.75	1.68	1.82	1.04
Phu-9. My mobile phone use increases day by day	730	83.82	102	11.71	39	4.48	1.61	1.55	1.67	0.90
Phu-10. The time allocated to social, personal or professional activities decreases because of my mobile phone	731	83.92	75	8.61	65	7.47	1.59	1.52	1.66	1.00

**Table 3:** Phubbing levels (n = 871).

Phubbing Levels	Range of Scores	Frequency (n)	Percentage (%)
Occasional phubbing	<14	101	11.6%
Frequent phubbing	14–24	586	67.3%
Risky phubbing	25–30	135	15.5%
Problematic phubbing	≥31	49	5.6%

**Table 4:** Percentage distribution of students according to prevalence levels of phubbing by dimension.

Dimension	Occasional Level	Frequent Level	Risky Level	Problematic Level
Attachment to the mobile phone	0.0%	78.3%	16.0%	5.7%
Communication disturbance	0.0%	71.1%	22.3%	6.7%
Smartphone obsession	12.3%	63.9%	17.7%	6.1%

**Table 5:** Means and standard deviations in phubbing dimensions according to gender.

	Attachment to the Mobile Phone		Communication Disturbance		Smartphone Obsession	
**Gender**	**Mean**	**SD**	**Mean**	**SD**	**Mean**	**SD**
Men	1.48	0.79	1.74	0.79	1.97	0.71
Women	1.33	0.68	1.69	0.72	2.12	0.79

**Table 6:** Means and standard deviations in phubbing dimensions according to grouped age.

	Attachment to the Mobile Phone		Communication Disturbance		Smartphone Obsession	
**Age**	**Mean**	**SD**	**Mean**	**SD**	**Mean**	**SD**
Younger than 20 years old	1.41	0.71	1.79	0.78	2.17	0.81
From 20 to 24 years old	1.40	0.73	1.72	0.75	2.11	0.78
From 25 to 29 years old	1.37	0.74	1.67	0.72	2.02	0.78
Older than 30 years old	1.28	0.66	1.53	0.68	1.92	0.75

**Table 7:** Means and standard deviations in phubbing dimensions according Internet connection time.

Daily Connection Frequency	Attachment to the Mobile Phone		Communication Disturbance		Smartphone Obsession	
	Mean	SD	Mean	SD	Mean	SD
Less than 1 h	1.18	0.61	1.48	0.64	1.71	0.69
Between 1 and 2 h	1.31	0.66	1.61	0.68	1.89	0.72
Between 2 and 3 h	1.36	0.68	1.68	0.69	2.00	0.76
Between 3 and 4 h	1.41	0.72	1.72	0.70	2.08	0.76
More than 4 h	1.53	0.80	1.84	0.81	2.26	0.81

**Table 8:** Means and standard deviations in the dimensions of phubbing according to academic performance.

Academic Performance	Attachment to the Mobile Phone		Communication Disturbance		Smartphone Obsession	
	Mean	SD	Mean	SD	Mean	SD
Low	2.38	1.7	2.08	1.26	1.94	0.88
Medium	1.5	0.83	1.69	0.77	2.14	0.87
High	1.32	0.66	1.7	0.73	2.1	0.74
Very high	1.2	0.46	1.68	0.68	1.86	0.76

## Data Availability

The data presented in this study are available on request from the corresponding authors.

## Abbreviations

The following abbreviations are used in this manuscript:

ANOVA	Analysis of Variance
CFA	Confirmatory Factor Analysis
CFI	Comparative Fit Index
DWLS	Diagonally Weighted Least Squares (estimator)
EFA	Exploratory Factor Analysis
FOMO	Fear of Missing Out
GFI	Goodness-of-Fit Index
ICT	Information and Communication Technologies
IFI	Incremental Fit Index
JASP	Jeffreys’s Amazing Statistics Program (statistical software)
KMO	Kaiser–Meyer–Olkin Measure of Sampling Adequacy
MANOVA	Multivariate Analysis of Variance
NFI	Normed Fit Index
OECD	Organisation for Economic Co-operation and Development
PS	Phubbing Scale
RMSEA	Root Mean Square Error of Approximation
SDT	Self-Determination Theory
SPSS	Statistical Package for the Social Sciences
SRMR	Standardized Root Mean Square Residual
TLI	Tucker–Lewis Index
UNESCO	United Nations Educational, Scientific and Cultural Organization
